# Value of laryngeal ultrasound in comparison with flexible laryngoscope in diagnosis of various laryngeal masses: a cross-sectional study

**DOI:** 10.1186/s43055-022-00904-y

**Published:** 2022-10-17

**Authors:** Amr A. Elfattah Hassan Gadalla, Khaled Mohamed Othman, Mo’men Ali Ameen Hamela, Abo El Magd Mohamed El Bohy

**Affiliations:** 1 Faculty of Medicine, Radiology Department, Cairo University, Cairo, Egypt; 2 Faculty of Medicine, Department of Otorhinolaryngology, Cairo University, Cairo, Egypt

**Keywords:** Laryngeal masses, Vocal cord, Laryngeal ultrasound, Flexible laryngoscopy

## Abstract

**Background:**

The term "vocal cord mass" refers to any abnormal growth of the vocal cords. This may include nodules, cysts, polyps, or cancers. Hoarseness of voice is considered the most common symptom of laryngeal lesions. Ultrasound imaging has evolved into a valuable and effective technique for evaluating the head and neck region, including the larynx's structure. Our aim is to evaluate laryngeal ultrasound as an alternative technique to the flexible laryngoscope in the diagnosis and assessment of various laryngeal masses.

**Results:**

The study included 30 males (57.7%) and 22 females (42.3%) who presented with hoarseness of voice. All patients had laryngeal ultrasonography, which was followed by flexible laryngoscopy. The most commonly affected cord was the left vocal cord (42.3%), followed by the right vocal cord (32.7%), and finally both of them (25%). The sensitivity of laryngeal ultrasonography was 88.5% compared to the gold standard flexible laryngoscopy (*p* value 0.031).

**Conclusions:**

The laryngeal ultrasonography is a highly successful technique in the diagnosis and assessment of various laryngeal masses and could be complementary to flexible laryngoscopy in many cases, especially when laryngoscopy is contraindicated, with relatively high sensitivity in the detection of laryngeal masses.

## Background

Laryngeal masses account for 80% of all benign lesions found. The etiology of benign vocal cord masses is typically complex with phono-trauma (cough and extreme loudness), trauma of the larynx (endotracheal intubation), and smoking. Patients primarily complained of hoarseness of voice. Presently, in clinical practice, vocal cord polyps are the most prevalent benign vocal cord lesions [1–3]. After skin cancer, laryngeal carcinoma is the second most frequent malignancy of the head and neck. The disease is responsible for 30 to 40% of all head and neck cancers, as well as 1 to 2.5% of all cancers in the human body. The most common type of laryngeal cancer is squamous cell carcinoma. Patients with a history of smoking account for almost 90% of cases, and the chances are even higher if they also have a history of excessive alcohol usage [4, 5]. Laryngoscopy, computed tomography (CT), and magnetic resonance imaging (MRI) are common diagnostic and staging tools for cancer of the larynx. Ultrasonography is commonly used to evaluate the involvement of cervical lymph nodes and soft tissues in patients with cancer of the larynx, but it is uncommon to evaluate the tumor itself and its invasion of the laryngeal structures due to the thyroid cartilage calcifications in adults and the interference of air within the laryngeal cavities [6].

However, attempts have been made to use ultrasonography (US) in this region to take advantage of its non-invasive and real-time imaging features and have noticed that ultrasonography has a similar role in the visualization of cancer larynx to CT, locating the primary tumor focus and evaluating the tumor spread inside and outside the larynx [6, 7].

Until now, laryngoscopy has been regarded as the primary approach for diagnosing VC lesions, despite the fact that this tool is not appropriate for some elderly patients, adult patients, and children. Because of its affordability, non-ionizing radiation, non-invasiveness, and real-time capabilities, ultrasonographic imaging has progressively become a very effective and powerful tool in assessing the head and neck areas, yet it is user-dependent [8, 9].

The main aim of this study is to evaluate the role of laryngeal ultrasound as an alternative to the flexible laryngoscope in the diagnosis and assessment of various laryngeal masses.

## Methods

This is a prospective cross-sectional cohort study accepted by the local ethical committee, including patients from the ENT department during the period from March 2021 to September 2021 referred to the ultrasound and Doppler unit at the radiology department.

The study involved patients who had hoarseness of voice. Full written consent was signed by all patients. Fifty-two patients were included, 30 males and 22 females, with an age range from 30 to 73 years.

Patients included are the patients with hoarseness of voice referred for blind examination by ultrasound, which revealed vocal cord pathology, including vocal cord polyps and other laryngeal masses.

Patients with other causes of hoarseness of voice, such as vocal cord paralysis and laryngitis, patients who have recently had neck surgery, patients who have recently had neck trauma, and patients with a tracheostomy were excluded.

### Imaging/Examination protocol

All patients had laryngeal US done then, followed on the same day by flexible laryngoscopy. The laryngeal US was done using the Toshiba Aplio 500 and General Electric Logic P6 and with a linear probe of (7.5–12 MHz frequency).

*Patient preparation* No preparation is required. There were no significant risk factors as regards the ultrasound examination in this study (a non-invasive procedure).

### Technique

*Laryngeal ultrasound* The patient lays supine with a little cushion supporting the shoulders and a slightly extended neck, and the gel was applied to the linear probe of the examination. A combined anterior and lateral approach laryngeal ultrasound examination was done. The anterior approach started with the identification of the hyoid bone. This is heavily calcified and forms the attachment for all the major strap muscles. Then the thyroid cartilage is located by looking just beneath the strap muscles. Then, scanning in the transverse plane to locate the vocal cords, the true vocal folds appear hypoechoic and the false cords appear hyperechoic. In the lateral approach, the transducer was positioned on the lateral surface of the larynx. Once the laryngeal landmarks are localized (true vocal cord, false vocal cord, and arytenoid cartilage), the view is kept up to date for continual monitoring. The vocal cords were assessed during hold breathing, which allowed for a better evaluation of the VC and their masses, and then during vowel phonation "a," "e,” or "i" by axial and/or sagittal scanning to examine the vocal cord mobility and function.

This examination was done by two expert radiology doctors with a minimum of three years of experience with calculated inter-reader agreement revealing about 96.1 % agreement.

*Flexible laryngoscope* The patient is sitting in front of the examiner. Nasal decongestants are used for mucosal vasoconstriction. The pharynx and larynx are anesthetized with lidocaine (4.0%). The laryngoscope's tip is inserted into the nose and moved lateral to the septum and medial to the inferior turbinate. Beyond the middle turbinate and down the nasal floor, the laryngoscope is moved posteriorly into the nose. Patients are instructed to breathe using their noses in order to distinguish the palate from the posterior nasal wall and allow the scope to enter into the oropharynx. The scope is passed lower and lower until the larynx and vocal folds are visible. Patients are instructed to sniff or breathe deeply through their noses. The patients are then instructed to speak "e" or "ah" to assess the function, mobility, and lesions of the VC and arytenoid cartilages. This promotes maximal VC abduction, allowing for ideal laryngeal assessment. This examination is done by an ENT specialist with five years of experience.

### Statistical analysis

The statistical analysis was fulfilled using the statistical package for the Social Sciences (SPSS) 22nd edition; numeric variables were given in mean ± standard deviation and were compared by the Mann–Whitney U test following normality testing. Categorical variables were presented in frequency and percentage and were compared via the Chi-square (*χ*^2^) test. A paired comparison of binomial data was made via the McNamara test. Any p-value less than 0.05 is regarded as statistically significant.

## Results

We did this study on 30 males (57.7%) and 22 females (42.3%). All patients underwent laryngeal ultrasonography followed by the flexible laryngoscope. Among the included patients, the mean age was 46.3 ± 12.6 years old. The most commonly affected cord was the left vocal cord (42.3%), followed by the right vocal cord (32.7%), and finally both of them (25%). Moreover, the anterior third of the vocal cords was affected in (44.2%) of the cases, followed by the middle part (30.9%), then the middle and anterior thirds (9.6%), and finally the posterior third (3.8%) (Table 1).

**Table 1:** The baseline characteristics of the patients included

		*N*	%
Age (mean ± SD)		46.3	12.6
Sex	Male	30	57.7%
	Female	22	42.3%
Side	Right	17	32.7%
	Left	22	42.3%
	Both cords	13	25.0%
Flexible laryngoscope	Negative	0	0.0%
	Positive	52	100.0%
Ultrasonography	Negative	6	11.5%
	Positive	46	88.5%
Site of lesion within vocal cord	Could not be assessed	6	11.5%
	Anterior third	23	44.2%
	Middle third	16	30.9%
	Anterior and middle thirds	5	9.6%
	Posterior third	2	3.8%

Pathology findings after surgical excision of the above-mentioned laryngeal masses revealed 19 cases of laryngeal cysts and 15 cases of polyps. Furthermore, laryngeal cancer was seen in 16 patients; 9 patients presented with masses and 7 patients presented with nodules. Just two patients had leukoplakia.

**Fig. 1:**
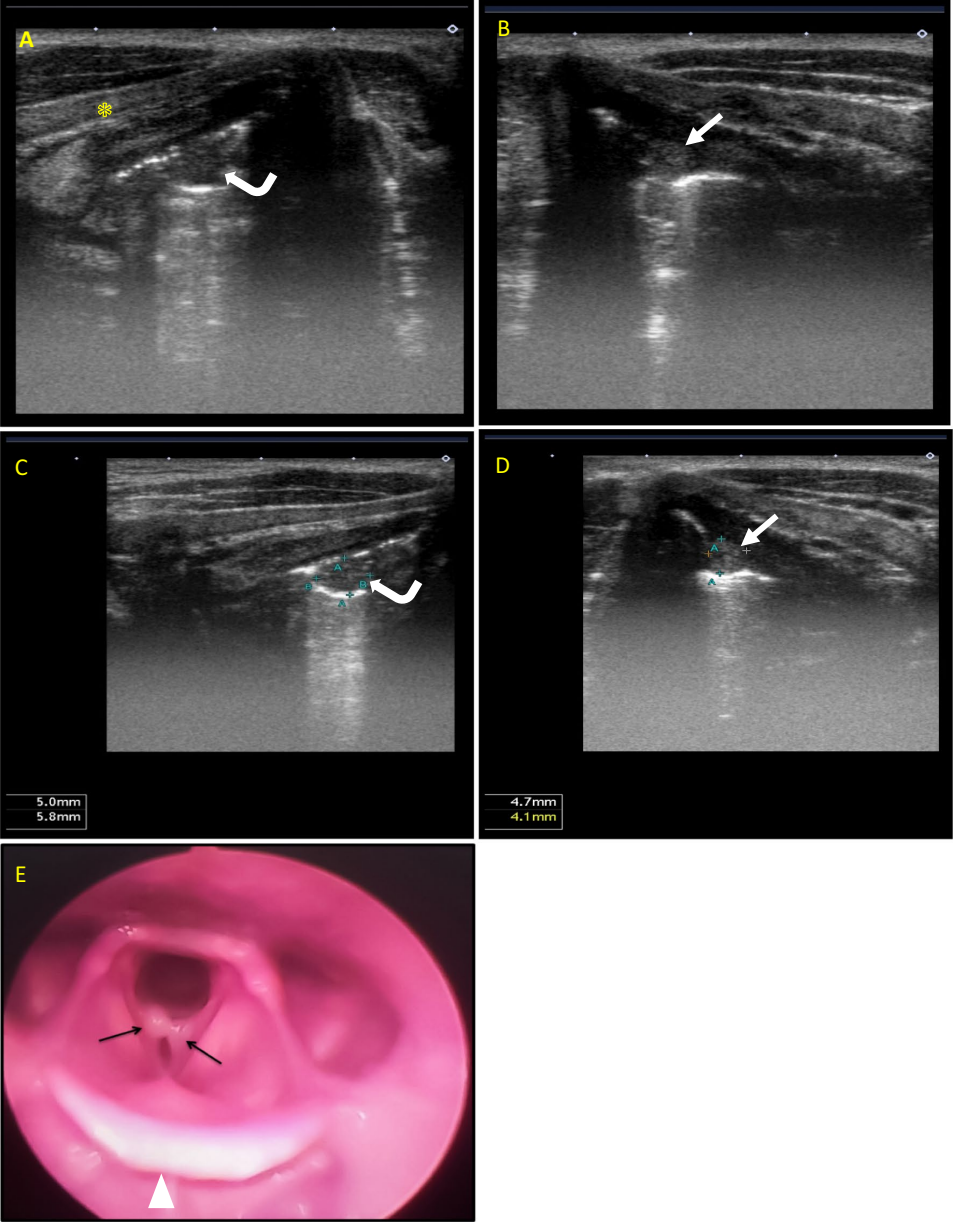
A 30-year-old heavy smoker, male patient presented with hoarseness of voice. **A** &** C** Laryngeal ultrasound right lateral axial views show a right VC hypoechoic small nodule (**curved arrows**) measuring about 5 × 5.8 mm with posterior acoustic enhancement (thyroid cartilage landmark with asterisk).** B** &** D** Laryngeal ultrasound left lateral axial views show left VC hypoechoic small lesion (**white arrows**) measures about 4.7 × 4.1 mm with posterior acoustic enhancement. The vocal cord nodules show internal echoes, and this raises the possibility of highly proteinaceous or hemorrhagic cysts.** E** Flexible laryngoscopy with the anterior is at the bottom of this figure (epiglottis marked by arrow-head), reporting bilateral vocal cord polyps (**black arrows**). Endoscopic removal of the vocal cord lesions and histopathology assessment revealed complicated cysts matching the sonographic findings as acoustic enhancement in ultrasound confirms that it is a cystic lesion rather than vocal polyps

**Fig. 2:**
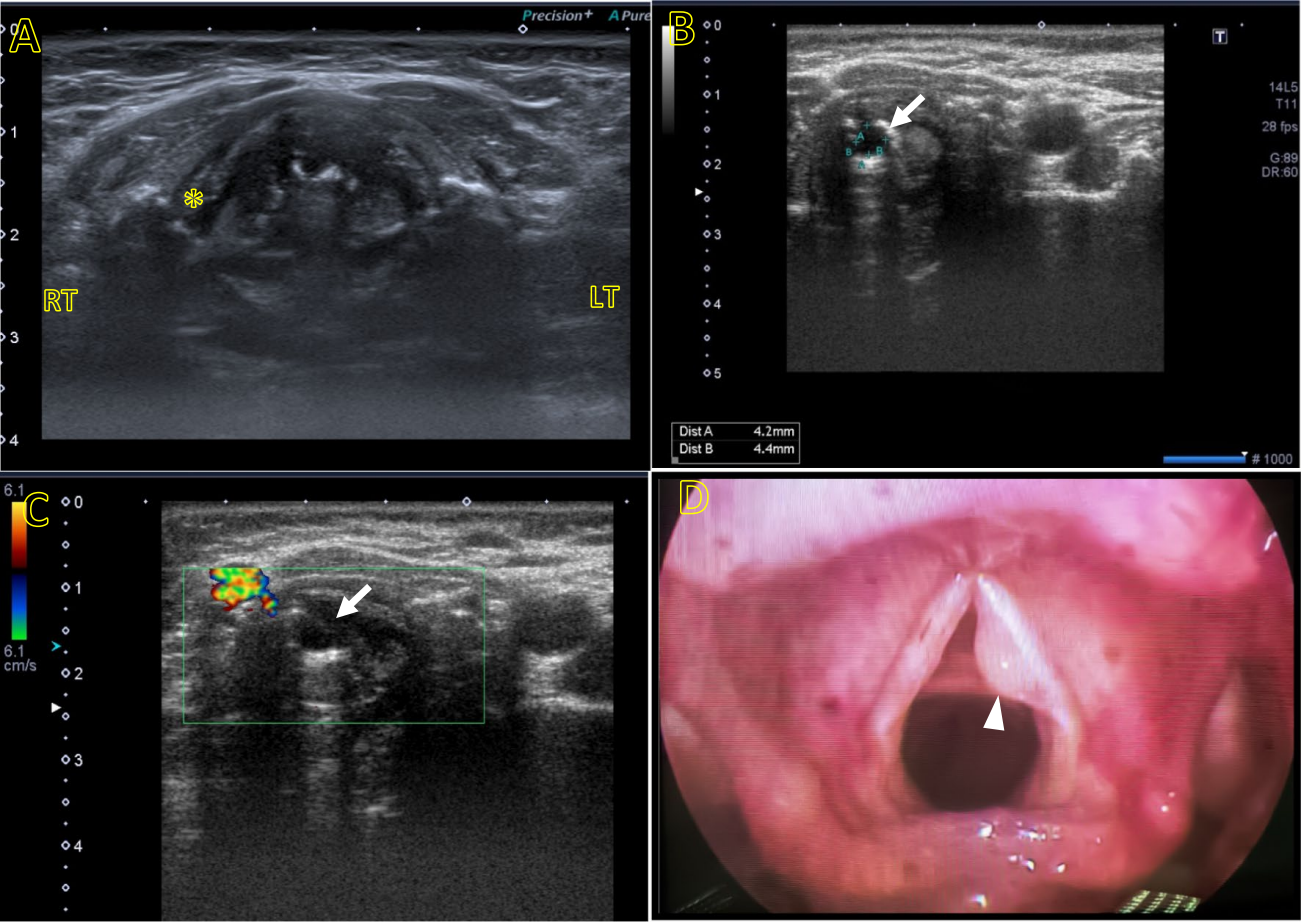
A 55-year-old female patient presented with hoarseness of voice and GERD. **A** Laryngeal ultrasound anterior axial view showing the anatomical landmarks along the level of the true vocal cord with thyroid cartilage seen (asterisk) and right/left landmarks on the image. **B** Left lateral axial view shows left VC anechoic lesion measures about 4 mm (arrow) with no color Doppler signal uptake could be traced **C** denoting vocal cord cyst. **D** Flexible laryngoscopy of the same patient shows a left VC cyst (arrow head), matching the sonographic diagnosis

In our study, combined anterior and lateral approaches to laryngeal ultrasound were adopted to visualize the structure of the larynx. However, in a few male patients (3 cases) with densely calcified thyroid cartilage, the lateral approach was sufficient to visualize the vocal cords (Figs. 1, 2).

There was a statically significant difference between the included patients based on the ultrasound findings described in (Table 2), with older age groups being more prone to false-negative results, with a mean age of 56.2 ± 8.9 years versus 45.1 ± 12.5 years (*p *= 0.033). False-negative results were all reported in males, which made a significant difference in ultrasound findings (*p* = 0.026).

**Table 2:** The comparison of baseline characters based on the ultrasound findings

		Ultrasonography				*P *value
		Negative		Positive		
		*N*	%	*N*	%	
Age		56.2	8.9	45.1	12.5	0.033*
Sex	Male	6	100.0%	24	52.2%	0.026**
	Female	0	0.0%	22	47.8%	
Side	Right	2	33.3%	15	32.6%	0.85**
	Left	2	33.3%	20	43.5%	
	Both cords	2	33.3%	11	23.9%	
Site of lesion	Not applicable	6	100.0%	0	0.0%	0.0001**
	Anterior third	0	0.0%	23	50%	
	Middle third	0	0.0%	16	34.8%	
	Anterior and middle thirds	0	0.0%	5	10.9%	
	Posterior third	0	0.0%	2	4.3%	

*Mann–Whitney u test

**Chi-square (*χ*^2^) test

**Fig. 3:**
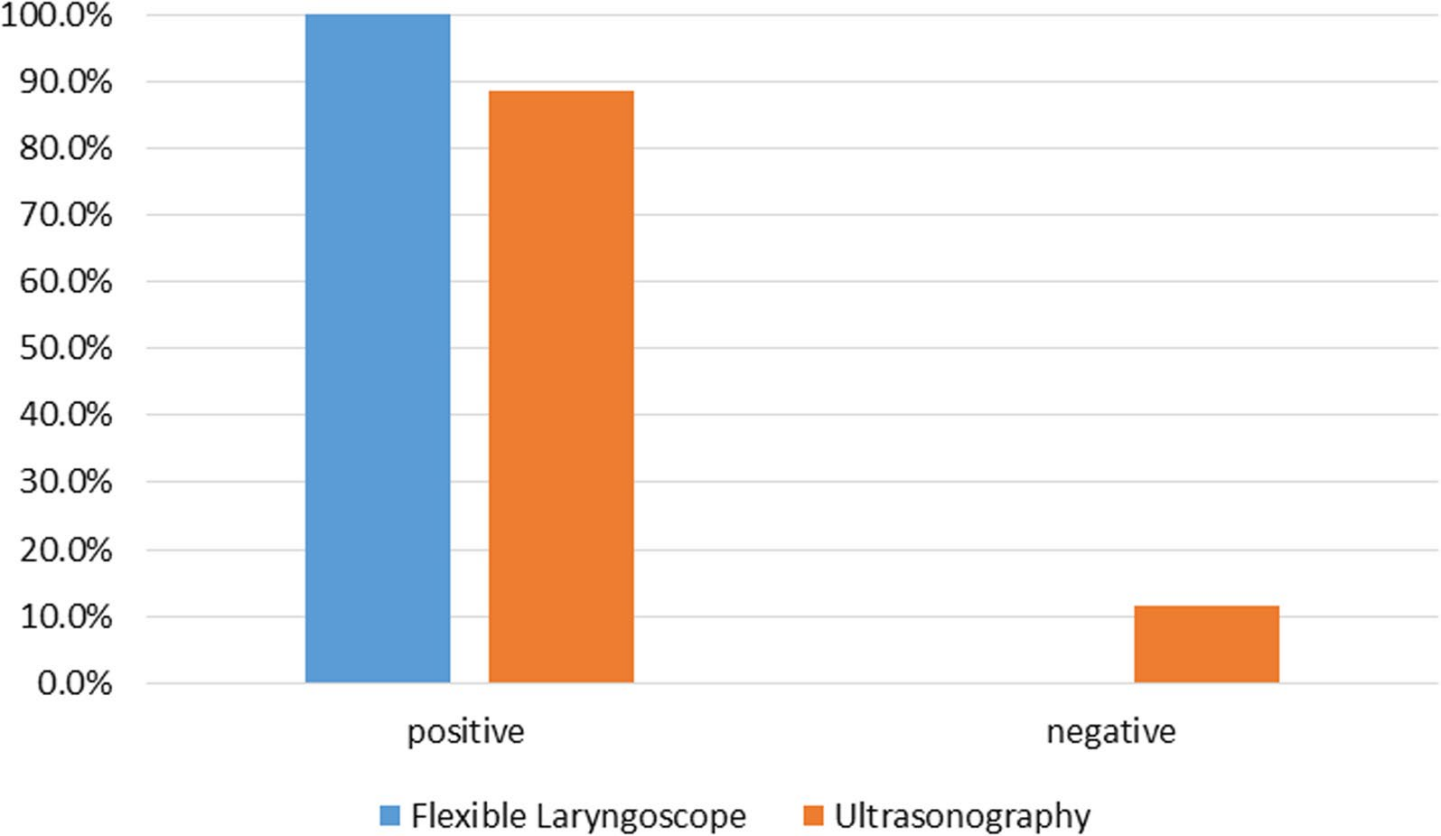
A bar chart showing the results of laryngeal ultrasound and flexible laryngoscopy

Paired comparison of laryngeal ultrasound and flexible laryngoscopy findings revealed that there was a significant difference between both modalities. The sensitivity of laryngeal ultrasonography was 88.5% compared to the gold standard flexible laryngoscopy (*p *= 0.031) (Table 3, Fig. 3).

**Table 3:** The paired comparison between findings of flexible laryngoscopy and laryngeal ultrasound

		Ultrasonography				*P *value
		Negative		Positive		
		*N*	%	*N*	%	
Flexible laryngoscope	Negative	0	0.0%	0	0.0%	0.031
	Positive	6	11.5%	46	88.5%	

McNamara test

## Discussion

Many studies have found that, when compared to the laryngoscope, laryngeal ultrasonography is less expensive, more tolerable, and more reliable in evaluating the function and lesions of the vocal folds. Over the last 40 years, laryngeal ultrasound has evolved, and several researchers have evaluated its utility. Some research has been published on the anatomical structures reported by laryngeal ultrasound [10–12]. Prasad et al [13] revealed that laryngeal ultrasonography can clearly view the soft tissues surrounding the upper airways, indicating that it has a wide range of clinical applications.

In the present study, paired comparison of laryngeal ultrasonography and flexible laryngoscopy findings showed a considerable difference between both modalities. The sensitivity of the flexible laryngoscopy was 100% versus 88.5% in laryngeal ultrasonography (*p *= 0.031). Ultrasound could not detect laryngeal lesions in 6 patients who were males and had TC calcifications, which hindered the detection of laryngeal lesions. This limitation is also described by Wenaas et al and Garvin et al studies [14, 15]. Laryngoscopy of those patients revealed bilateral leukoplakia in 2 cases, right vocal cord nodules in 2 cases, left vocal cord nodule in one case, and finally a left supraglottic mass in the last case.

Regarding the results of the flexible laryngoscopy of those 6 patients who were negative by the US, leukoplakia is just a thickening of the VC and is not an exophytic lesion, so it could not be visualized by ultrasound. The case of the left supraglottic mass could not be visualized by the US because the densely calcified shadowing thyroid cartilage completely masked the laryngeal structure, and this agrees with Wenaas et al. and Garvin et al. [14, 15]. Finally, due to their small size, the three cases of VC nodules could not be detected by the US, which agrees with Schade et al. [16], who attempted to demonstrate whether the results of the laryngeal US are better than the laryngoscope's or whether ultrasonography offers any additional advantages. They reported that US is useful in diagnosing larger laryngeal masses but not in detecting small nodules.

According to the ultrasound findings, as previously mentioned, comparison of baseline characteristics revealed a significant difference among the included patients, with older age groups being more susceptible to false-negative results (p = 0.033), and the false-negative results being all reported in males. This might be related to the fact that males were more likely than females to have thyroid cartilage calcifications.

Our results agree with Wang and his co-workers [10], who conducted a cross-sectional study on 87 individuals who had postoperative pathology that revealed VC polyps. The rate of detection of VC polyps by the laryngeal US was 88.0% for all laryngeal lesions. Also, our results are in line with the results reported by Xia et al. [6], who studied 72 patients using laryngeal US but used CT scan as the gold standard and found that ultrasonography had a sensitivity of 87.5% compared to CT in the detection of laryngeal lesions but that ultrasound had a significantly higher specificity in the assessment of paraglottic space involvement. Furthermore, our findings are more sensitive than those of Sadek et al. [17], who found a lower sensitivity (78.5%) for ultrasonographic detection of bilateral VC masses compared to laryngoscopy but a higher sensitivity (93.7%) in cases of unilateral VC masses. The authors observed that laryngeal ultrasound has high sensitivity for single vocal cord nodules but a much lower sensitivity for bilateral vocal cord nodules [17, 18].

**Fig. 4:**
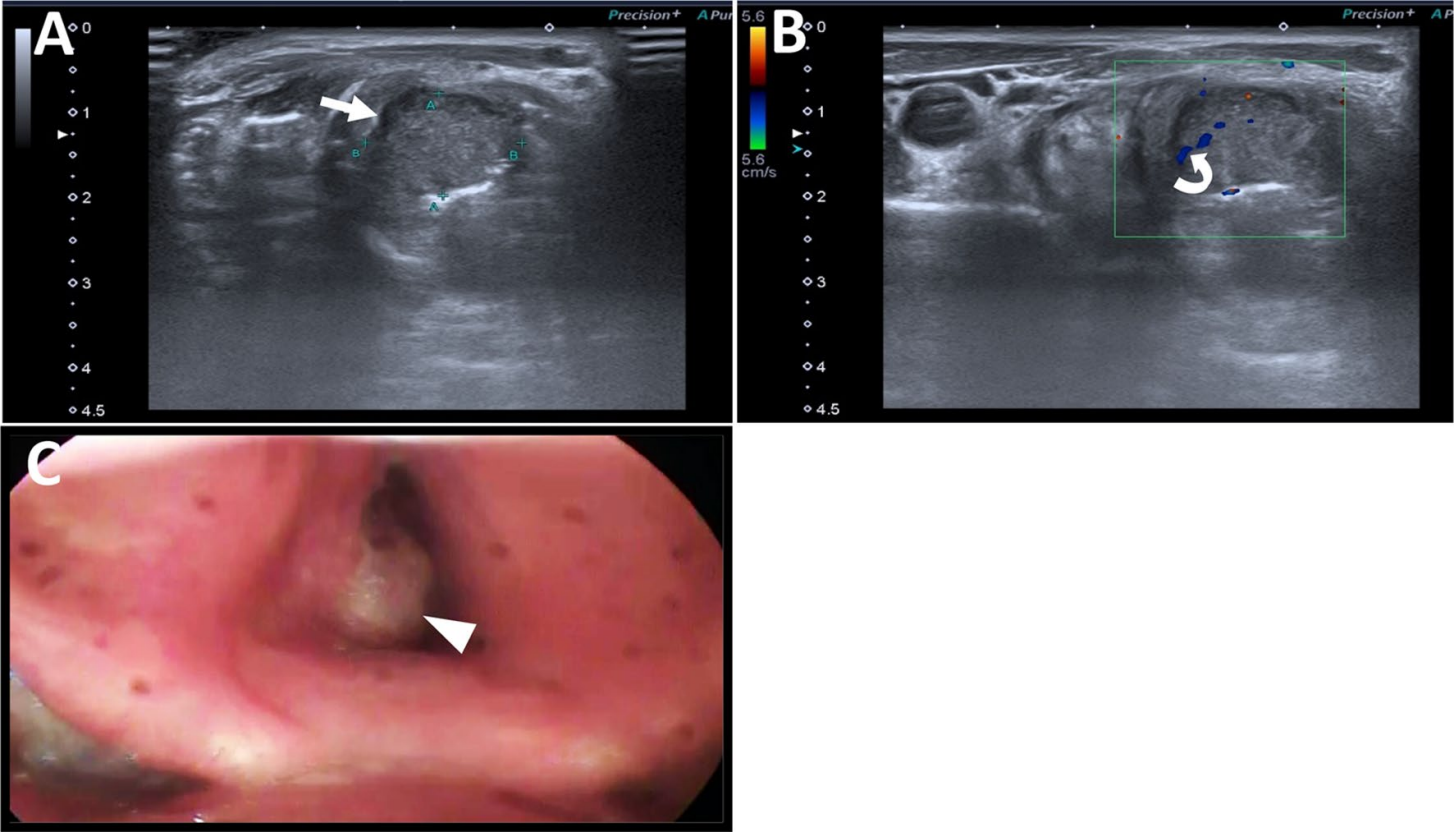
A 73-year-old male heavy smoker presented with hoarseness of voice, dysphagia, and stridor. **A** Laryngeal ultrasound right lateral axial view with right thyroid cartilage (asterisk) as anatomical landmark, shows a large soft tissue isoechoic mass lesion (arrow) almost completely replacing the right VC with ill-defined lateral portion of the right thyroid cartilage, indicating invasion. No related cervical LNs were detected. **B** Colored Doppler sonographic images revealed that the lesion has an internal color signal (curved arrow) denoting its hypervascularity. **C** Flexible laryngoscopy of the same patient shows the lesion in the posterior part of the right vocal cord (arrow head). The anterior is at the bottom of this figure. The flexible laryngoscopy confirmed the sonographic findings, yet it is limited in estimation of the proper size and extension of the subglottic component

In our work, we noted that ultrasonography is superior to laryngoscopy in the assessment of size and extension of masses, especially the subglottic and lateral extensions, as well as in the assessment of invasion of the laryngeal skeleton and extra laryngeal extensions as presented in our cases. Additionally, in our current study, we identified numerous cases of VC cysts with anechoic or turbid contents, well-defined outlines, and prominent posterior acoustic enhancement. Six of these cases were misdiagnosed by laryngoscopy as polyps, demonstrating that ultrasonography is more accurate than laryngoscopy in identifying such lesions. This agrees with Gomaa et al. [19], (Fig. 4).

**Fig. 5:**
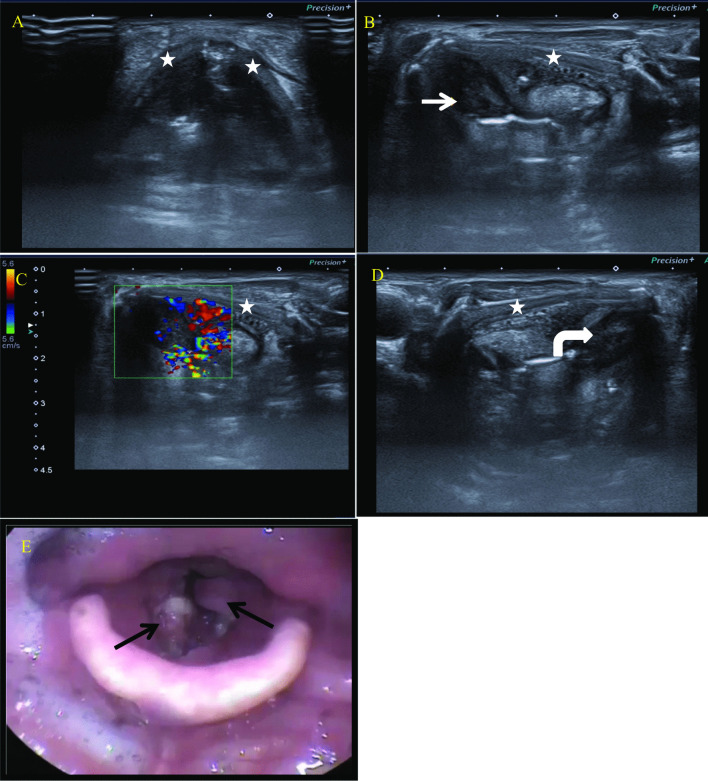
A 68-year-old heavy smoker, male patient presented with hoarseness of voice and stridor. **A** Laryngeal ultrasound anterior axial view shows the anatomical landmarks along the level of the true vocal cord with thyroid cartilage (marked by a star). **B** Laryngeal US, left lateral axial view, shows left glottic and supraglottic mass lesion (arrow) with ill-definition of the posterior aspect of the left thyroid lamina (marked by a star) near the midline, denoting erosion/invasion. **C** Colored Doppler assessment shows internal hypervascularity of the mass lesion. **D** Laryngeal US, right lateral axial view, shows right glottic and supraglottic mass lesion (curved arrow). **E** Flexible laryngoscopy of the same patient shows bilateral glottic and supraglottic mass lesions (black arrows). The anterior is at the bottom of this figure

Our observations are in agreement with those of Gomaa et al. [19], who reported that ultrasonography is more accurate than laryngoscopy in the detection of small subglottic swellings that hide under the vocal cords and that ultrasonography has advantages over the laryngoscope in detecting masses involving paraglottic, preepiglottic, and TC infiltration. Also, similar to the results reported by Xia et al. [6], (Fig. 5).

We had encouraging results regarding ultrasonographic assessment of laryngeal masses, and we expect that with time, with increased research in the field of laryngeal ultrasonography, further advances and refinement of the ultrasound machine, and with growing experience in the field, laryngeal ultrasonography is expected to become complementary to laryngoscopy in the diagnosis of various laryngeal masses.

### This study has some limitations

First, the decreased patient flow to our hospital as a result of the COVID-19 quarantine had an impact on sample size. Laryngeal ultrasound is an operator-dependent technique that depends on the operator’s experience and other factors like the machine quality and the patient’s compliance and cooperation. To reduce bias, the inter-observer agreement percentage is calculated, and the participating sample is examined with a single machine. Comparison with the laryngoscopy limits examination of a normal healthy control group. Finally, using new technique in the examination of laryngeal masses under research makes results limited to the available machine software.

## Conclusions

Laryngeal ultrasonography has a valuable role in the detection and screening of the structure of the larynx and different laryngeal masses comparable to laryngoscope. It can accurately assess the size and extensions of laryngeal masses better than the laryngoscope. Laryngeal ultrasound efficacy is limited in presence of dense thyroid cartilage calcifications; nevertheless, laryngeal ultrasound is a cheap, readily available method for evaluating the laryngeal skeleton and screening for detection of laryngeal masses. In addition, laryngeal ultrasound can complement laryngoscope in the work-up of patients with hoarseness of voice and suspected laryngeal masses.

## Data Availability

All the datasets used and analyzed in this study are available with the corresponding author on reasonable request.

## Abbreviations

AC: Arytenoid cartilage
CT: Computed tomography
FL: Flexible laryngoscopy
FVC: False vocal cord
LT: Left
MRI: Magnetic resonance imaging
RT: Right
SCC: Squamous cell carcinoma
SPSS: Statistical Package for Social Science
TC: Thyroid cartilage
TVC: True vocal cord
Us: Ultrasound
VC: Vocal cord
